# *BmElmo* is a factor for inhibiting *Autographa Californica* nucleopolyhedrovirus infection in silkworm, *Bombyx mori*

**DOI:** 10.3389/fimmu.2025.1495672

**Published:** 2025-04-02

**Authors:** Zhi-sheng Wang, Wen-jing Yu, Xin-yi Ding, Zhan-peng Lu, Sheng Qin, Xia Sun, Xue-yang Wang, Mu-wang Li

**Affiliations:** ^1^ Jiangsu Key Laboratory of Sericultural and Animal Biotechnology, School of Biotechnology, Jiangsu University of Science and Technology, Zhenjiang, China; ^2^ Key Laboratory of Silkworm and Mulberry Genetic Improvement, Ministry of Agriculture and Rural Affairs, Sericultural Scientific Research Center, Chinese Academy of Agricultural Sciences, Zhenjiang, China

**Keywords:** *BmElmo*, AcMNPV, *Bombyx mori*, baculovirus, JNK pathway

## Abstract

*Autographa californica* nucleopolyhedrovirus (AcMNPV) is a DNA virus with multiple host domains, and elucidating the mechanisms of its interactions with silkworms is crucial for its widespread use. Identifying key antiviral genes and analyzing their functions is an urgent task currently. Therefore, the identification and study of host genes associated with AcMNPV invasion is of great significance in solving the issue. Engulfment and cell motility (*Elmo*) is an identified viral infection-associated gene primarily involved in the regulation of cell motility and essential for phagocytosis and immune responses. However, its function in the silkworm response to viruses is still unclear. In this study, the sequence of *BmElmo* was analyzed first. It has a CED-12 functional domain that has been highly conserved among different species. Its expression peaks during the silkworm pupal stage, followed by the moth stage. Among various tissues, *BmElmo* expression is highest in the gonads, followed by the silk glands. *BmElmo* exhibits differential expression between resistant and susceptible strains. AcMNPV replication increased significantly after *BmElmo* knockdown in BmN cells, and decreased significantly after *BmElmo* overexpression. Furthermore, the expression of *Janus kinase* (JNK) pathway–related genes downstream of *BmElmo* showed altered expression that correlated positively with the expression of *BmElmo*. Hence, *BmElmo* may inhibit AcMNPV replication in the silkworm by activating the JNK pathway. The results of this study bridge the gap in understanding the role of *Elmo* genes in insect immunity and provides a theoretical reference for studying the interaction between insects and baculoviruses.

## 1 Introduction

The silkworm *Bombyx mori* (Lepidoptera: Bombycidae) serves as a model insect within the Lepidoptera order and is widely utilized in biological research, which makes it a significant insect in the realms of biology and agriculture (1). *Autographa californica* nucleopolyhedrovirus (AcMNPV) is a baculovirus with a double-stranded DNA genome (2), which has a wide range of host domains and therefore a wide range of applications. The silkworm and AcMNPV are good models for studying the interactions between hosts and baculoviruses (3), but fewer studies have been conducted on the functional genes of host antiviral resistance, resulting in the mechanisms of interactions that remain unclear. Therefore, there is an urgent need to identify the key antiviral genes and to analyze their functions.

*ELMO* was first discovered in the nematode *Caenorhabditis elegans* as a novel phagocytosis-related gene (4). Its functional domain, CED-12, plays an important role during cell migration and phagocytosis in *C. elegans*, *Drosophila*, and mammals (5, 6). In mammalian cells, there are three *Elmo* isoforms (7): *Elmo1*, *Elmo2*, and *Elmo3*. *Elmo* is an evolutionarily conserved protein and has no obvious catalytic region in its protein structure (8, 9). *Elmo1* can form a complex with the dedicator of cytokinesis (Dock) protein and act as a guanine nucleotide exchange factor (GEF) to activate Rac, thereby initiating the related signaling pathways downstream of Rac (10, 11), promoting cytoskeletal rearrangement and integrin-mediated phagocytosis and regulating cell migration (8, 12). In *Drosophila*, the CED-12 domain activates the JNK pathway to regulate ovarian nurse cell death and removal (13). In mice, *Elmo1* regulates chemotaxis and the adhesion of neutrophils defects *in vivo* and *in vitro* by activating Rac, thereby affecting immunity (14, 15). However, no research has linked Elmo to viral infections, nor have we seen studies of its role in silkworms. In our previous transcriptome analysis, we found that *BmElmo* expression varies significantly among silkworm strains with different resistance to AcMNPV infection (16), yet the underlying mechanism remains unclear.

In this study, the function of *BmElmo* in AcMNPV infection was examined by knocking down and overexpressing this gene in BmN cells (a silkworm ovary cell line) and the potential downstream pathway. The findings offer valuable insights into the molecular mechanisms underlying the interaction between lepidopterans and baculoviruses.

## 2 Materials and methods

### 2.1 Silkworms and AcMNPV

The p50 and C108 silkworm strain was bred at the Key Laboratory of Sericulture within the School of Life Science at Jiangsu University of Science and Technology. p50 is a susceptive strain of AcMNPV with a half lethal concentration (LC_50_) of 4.5×10^4^ pfu/mL, and C108 is a resistant strain of AcMNPV with a LC_50_ more than 6.2×10^8^ pfu/mL. The larvae were provided with fresh mulberry leaves and kept at 26 ± 1°C, 75% ± 5% relative humidity, and a 12-h photoperiod. The temperature was reduced to 24 ± 1°C during the final two instars, while the other conditions remained unchanged.

Budded AcMNPV with enhanced green fluorescent protein (BV-eGFP) is maintained in our laboratory. The titer of BV-eGFP was calculated as described by Li et al. (17).

### 2.2 Sample preparation

On the 1st day of 5th instar, each silkworm larva was injected with 2.0 μL of culture medium containing BV-eGFP at a concentration of 1.0 × 10^8^ plaque-forming units (pfu)/μL. The control group was injected with 2.0 μL of culture medium. Various tissues, including the midgut, fat body, hemolymph, and the Malpighian tubule, were collected 36 h after BV-eGFP injection. The samples were rapidly frozen in liquid nitrogen, immediately ground into a powder, and stored at -80°C until use. BmN cells were transfected with 2 ng of DNA/RNA or treated with 5.0 μL of culture medium containing BV-eGFP (1.0× 10^8^ pfu/μL) per well, and then collected at 24, 48, and 72 h post-transfection or BV-eGFP treatment. After centrifugation at low speed for 5 min and discarding the culture medium, phosphate-buffered saline (PBS) was added to wash the cells. Then, the cells were centrifuged at low speed for 5 min. The supernatant was discarded, and the cells were washed again. After the final wash, the cell pellet was frozen in liquid nitrogen, immediately ground into a powder, and then stored at -80°C until use.

### 2.3 Bioinformatics analysis

The *BmElmo* nucleic acid sequence (GenBank ID: LOC101744642) and its homologous sequences in other species were retrieved from the National Center for Biotechnology Information (NCBI) website (http://www.ncbi.nlm.nih.gov/). The functional domain of *BmElmo* was predicted using the online SMART tool (http://smart.embl-heidelberg.de/). The homologous nucleic acid sequence of *BmElmo* was analyzed using the DNAMAN 8.0 software, and a neighbor-joining tree was constructed using the MEGA-X software.

### 2.4 RNA extraction and complementary DNA synthesis

BmN cells and silkworm tissues were lysed with the TransZol reagent (Transgen, Beijing, China). Then, RNA was isolated by chloroform extraction, purified by the isopropanol precipitation, and dissolved in diethyl pyrocarbonate (DEPC)-treated water. The purity of the isolated RNA was assessed based on the optical density (OD) 260/280 ratio. The RNA concentration was measured using a NanoDrop 2000 spectrophotometer (Thermo, Waltham, USA), and its integrity was verified by 1% agarose gel electrophoresis. A total of 1.0 µg of RNA was subjected to reverse transcription with the PrimeScript™ RT reagent kit (Takara, Kusatsu, Japan) according to the manufacturer’s instructions.

### 2.5 Real-time quantitative polymerase chain reaction

The relative expression of the genes of interest was detected using RT-qPCR. The specific primers were designed using the NCBI website and are presented in **Table 1**. Each reaction comprised 1 µL of cDNA, 1 µL of primer mix, 3 µL of double-distilled water (ddH_2_O), and 5 µL of 2× NovoStart^®^ SYBR qPCR SuperMix Plus. The standard cycling protocol was 95°C for 5 min, followed by 40 cycles of denaturation at 95°C for 10 s and annealing at 60°C for 30 s, and finally a melting curve was generated at 95°C for 15 s, 60°C for 1 min, and 95°C for 15 s. To reduce experimental error, all samples underwent three repeats. The expression level of each gene was calculated using the 2−ΔΔCT method. *Bombyx mori* glyceraldehyde-3-phosphate dehydrogenase (*BmGAPDH*) was used as internal references to normalize the gene expression.

**Table 1:** List of the primers used for RT-qPCR.

Primer names	Forward primer (5’-3’)	Reverse primer (5’-3’)	Note
BmElmo	ggggtaccATGATAATTTACAATATTTGGACAG	cggaattcTTAAACTATATCTACCTCAATGTTTAT	Gene amplification
BmGAPDH	CCGCGTCCCTGTTGCTAAT	CTGCCTCCTTGACCTTTTGC	Reference gene
BmElmo	TTTCGAGGAATGGGCTTGCT	TGTAGGGTGTAGTGAGTGGCT	Interest gene
Lef3	CAAACGCGTTGCTTCGTACA	TGCTCGAGTCGGAAGAGGTA	Virus detect
BmJun	GAGACCACCTTCTATGACG	AATCTGGCGAGGAGAGAAC	JNK pathway
BmJnks	TGGACGCTAATCTGTGCCAA	CAGATCCCGATGAATGATTCCG	JNK pathway
BmFosS	CCTCCAGGAAGTTTACACG	TTTTCGGTCGACGGTGTTG	JNK pathway
BmActinA1	TCCTCCGTCTGGACTTGGC	CGATTTCCCTCTCAGCGGT	Phagocytosis
BmTet	CAGCGTCCTCCTCTTCACCT	CCTCGTCTGCGTTAGCGTC	Phagocytosis

### 2.6 Construction of the pIZT-mCherry-BmElmo overexpression vector

Using p50 testis cDNA as a template, the open reading frame (ORF) fragment of *BmElmo* was amplified using *BmElmo* primers (**Table 1**). The purified ORF was ligated into the pMD-19T vector and subsequently sent to Sangon (Shanghai, China) for sequencing. The pMD-19T-BmElmo and pIZT/V5-His-mCherry vectors were digested with Kpn*I* and EcoR*I* (Takara), and the correctly sequenced ORF was ligated into the overexpression vector using T4 DNA ligase (Takara). After sequencing validation, the pIZT/V5-His-mCherry-BmElmo vector was stored at -80°C until use.

### 2.7 Synthesis of small interfering RNA

siRNA targeting the functional domain of *BmElmo* was designed using the BLOCK-iT™ RNAi designer website (https://rnaidesigner.thermofisher.com/), which was used to knock down the expression of *BmElmo* in BmN cells. siRFP served as a negative control. The primer sequences are shown in **Table 2**. siRNAs were synthesized using the *In Vitro* Transcription T7 Kit (Takara) according to the manufacturer’s instructions. Following purification, the high-quality siRNA was stored at -80°C for future use.

**Table 2:** Sequences of the primers used to synthesize siRNAs.

Primer names	Sequence (5’-3’)
BmElmo-1 Olig-1	GATCACTAATACGACTCACTATAGGGCAGTTTGTTCCTAACAACTTTCACATT
BmElmo-1 Olig-2	AATGTGAAAGTTGTTAGGAACAAACTGCCCTATAGTGAGTCGTATTAGTGATC
BmElmo-1 Olig-3	AACAGTTTGTTCCTAACAACTTTCACACCCTATAGTGAGTCGTATTAGTGATC
BmElmo-1 Olig-4	GATCACTAATACGACTCACTATAGGGTGTGAAAGTTGTTAGGAACAAACTGTT
BmElmo-2 Olig-1	GATCACTAATACGACTCACTATAGGGCATACATGTTCAATGCCAAGCAATATT
BmElmo-2 Olig-2	AATATTGCTTGGCATTGAACATGTATGCCCTATAGTGAGTCGTATTAGTGATC
BmElmo-2 Olig-3	AACATACATGTTCAATGCCAAGCAATACCCTATAGTGAGTCGTATTAGTGATC
BmElmo-2 Olig-4	GATCACTAATACGACTCACTATAGGGTATTGCTTGGCATTGAACATGTATGTT
RFP-Olig-1	GATCACTAATACGACTCACTATAGGGGCACCCAGACCATGAGAATTT
RFP-Olig-2	AAATTCTCATGGTCTGGGTGCCCCTATAGTGAGTCGTATTAGTGATC
RFP-Olig-3	AAGCACCCAGACCATGAGAATCCCTATAGTGAGTCGTATTAGTGATC
RFP-Olig-4	GATCACTAATACGACTCACTATAGGGATTCTCATGGTCTGGGTGCTT

### 2.8 BmN cell culture and transfection

BmN cells were cultured at 27°C. Each 50 mL of cell culture medium comprised 44.5 mL of TC-100 (AppliChem, Gatersleben, Germany), 5 mL of fetal bovine serum (FBS; Thermo Fisher Scientific, New York, NY, USA), and 0.5 mL of dual antibiotics (penicillin and streptomycin). The culture medium of Sf9 cells was Sf-900TM (Thermo Fisher Scientific, New York, NY, USA). BmN cells were transfected with siRNA and the overexpression vector using a transfection reagent (NEOFECT, Beijing, China) according to the manufacturer’s instructions. Each 60 mm dish was transfected with 4.0 µg of plasmid or siRNA. The transfection solution for 2 mL of culture medium comprised 2 μg of DNAor RNA, 2 μL of transfection reagent (NEOFECT), and 100 μL of TC-100 solution; it was prepared 30 min before transfection. The transfection efficiency was measured at different time points. The expression level of BmElmo was detected at 24, 48, and 72 h after transfection.

### 2.9 Statistical analysis

All statistical analyses were performed using GraphPad Prism 8.0.1 (GraphPad Software, San Diego, CA, USA). The data are presented as the mean ± standard error of the mean of three biological replicates, and each biological replicate was derived from samples of different individuals. Student’s t-test was used to compare two groups of normally distributed data. The Kruskal–Wallis test was used to analyze the data that did not meet the normality requirement. Statistically significant differences between samples were analyzed using one-way ANOVA test. Significance is indicated by an asterisk: * *P* < 0.05, ** *P* < 0.01, and *** *P* < 0.001; ns means not significant.

## 3 Results

### 3.1 Bioinformatics analysis of *BmElmo*

**Figure 1:**
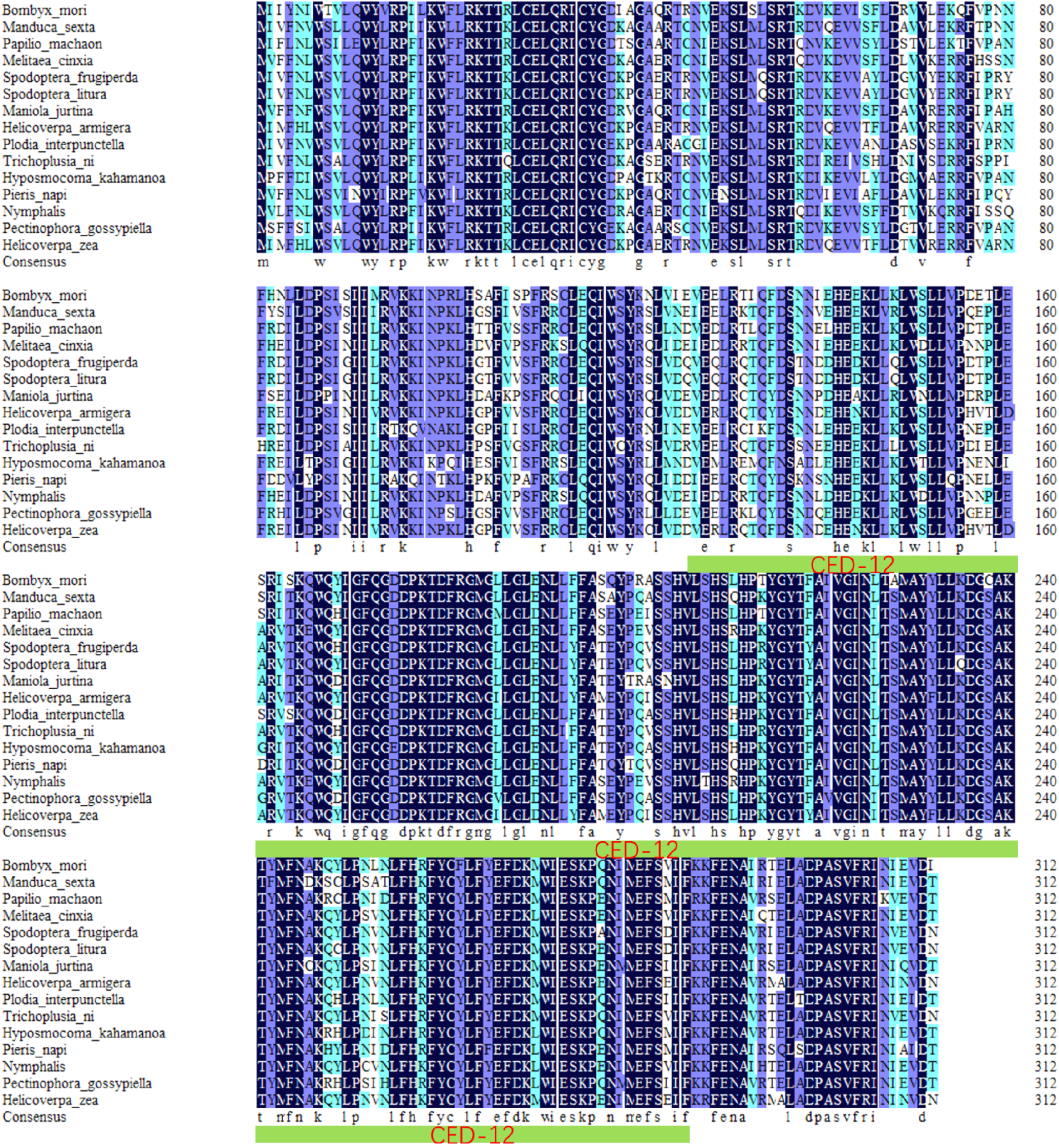
Analysis of BmElmo sequence conservation. A total of 15 species were compared. The green color with a thick underline indicates the functional domains of BmElmo. Conserved amino acid sequences are shown with a black background.

*BmElmo* (GenBank ID XM_004923980.4) has a full length of 3,725 base pairs (bp) and a coding sequencing (CDS) length of 936 bp, encoding a protein of 313 amino acids. Based on functional domain analysis of *BmElmo* using the SMART website, there is a CED-12 domain spanning amino acids 125 to 286. Additionally, protein sequence homology alignment analysis showed that *BmElmo* has a sequence conservation of 86.43% (**Figure 1**).

**Figure 2:**
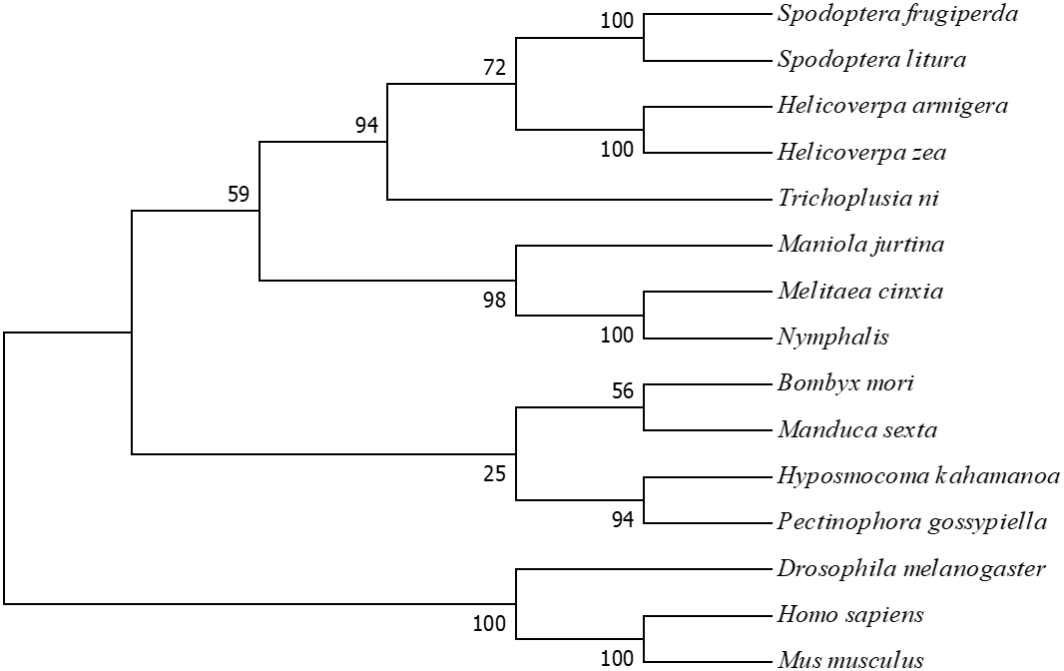
Phylogenetic tree of ELMO among 15 species. The bootstrap values from 1000 replicates are shown on each branch. The branch length represents the evolutionary distance.

An evolutionary tree has been constructed by neighbor joining method in the MEGA-X software with the identified homologous Elmo protein sequences from various species, including *B. mori*, *Drosophila melanogaster*, *Manduca sexta*, and *Melitaea cinxia* (**Figure 2**).

### 3.2 Spatiotemporal expression pattern of *BmElmo*

**Figure 3:**
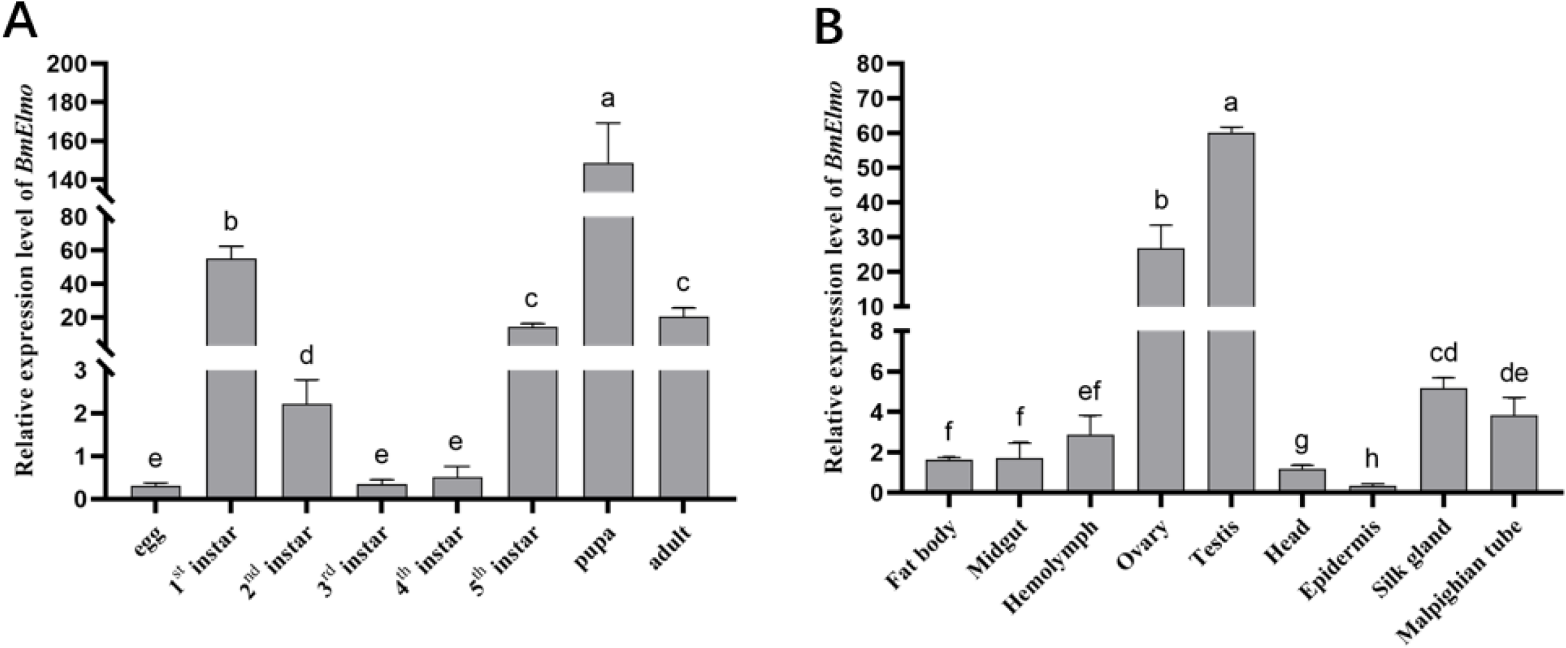
Analysis of *BmElmo* relative expression across various tissues and developmental phases based on RT-qPCR. **(A)** *BmElmo* expression at different developmental stages. **(B)** *BmElmo* expression in various tissues. *BmGAPDH* was used to normalize the data that were presented as the mean ± standard error of three replicates. Statistically significant differences between sample were analyzed using one-way ANOVA test. *P*<0,05 is indicated using different letters a, b, c, d, etc.

The relative expression of *BmElmo* across various developmental stages of the p50 strain and in different tissues of 5th instar larvae have been examined by RT qPCR. *BmElmo* expression was highest during the pupal stage, and there was notable expression for the 1st instar and adult stages. The egg stage presented the lowest expression (**Figure 3A**). Among the various tissues, *BmElmo* expression was highest in the testis, followed by the ovary and silk gland, while the epidermis presented the lowest expression (**Figure 3B**).

### 3.3 *BmElmo* expression changes in immune-related tissues after AcMNPV infection

**Figure 4:**
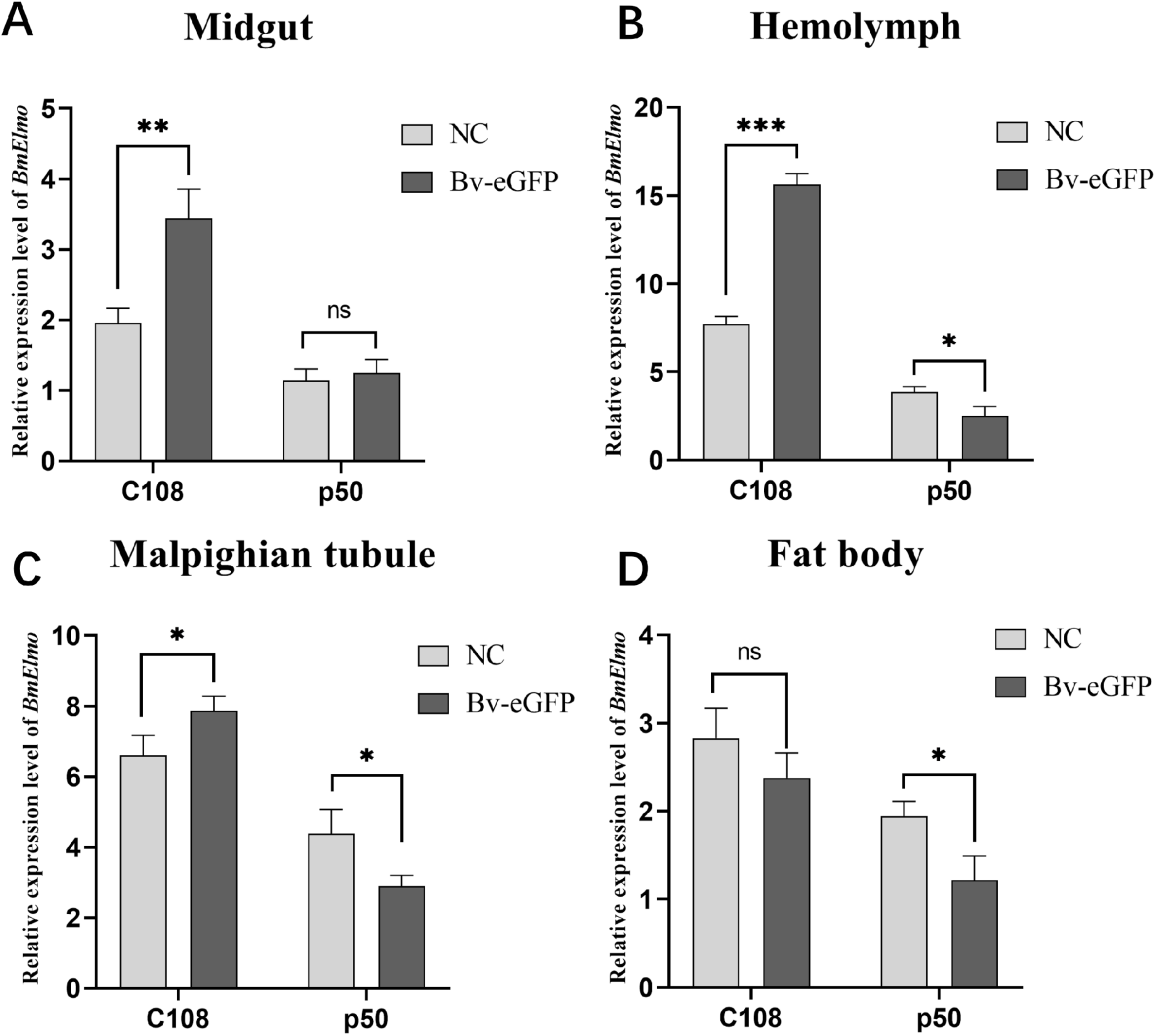
Analysis of *BmElmo* relative expression in various tissues of the susceptible (p50) and resistant (C108) strains after AcMNPV infection based on RT-qPCR. **(A)** Midgut, **(B)** Hemolymph, **(C)** Malpighian tubule, **(D)** Fat body. *BmElmo* expression was detected 36 h after AcMNPV infection. *BmGAPDH* was used to normalize the data that were presented as the mean ± standard error of three replicates. Asterisks represent significant differences. * *P* < 0.05, ** *P* < 0.01, and *** *P* < 0.001; ns indicates not significant.

The relationship between *BmElmo* and AcMNPV has been analyzed based on *BmElmo* expression in four immune-related tissues (hemolymph, midgut, Malpighian tubules, and fat body) from a susceptible strain (p50) and a resistant strain (C108) 36 h after AcMNPV infection. The silkworms in the control group were injected with same volume of SF-900tm insect culture medium. *BmElmo* expression in the midgut, hemolymph, and Malpighian tubule of the C108 strain was significantly elevated compared with the control group, while in the p50 strain, *BmElmo* expression in the Malpighian tubule, hemolymph, and fat body was notably decreased relative to the control group (**Figure 4**).

### 3.4 *BmElmo* knockdown inhibits BV-eGFP infection *in vitro*

**Figure 5:**
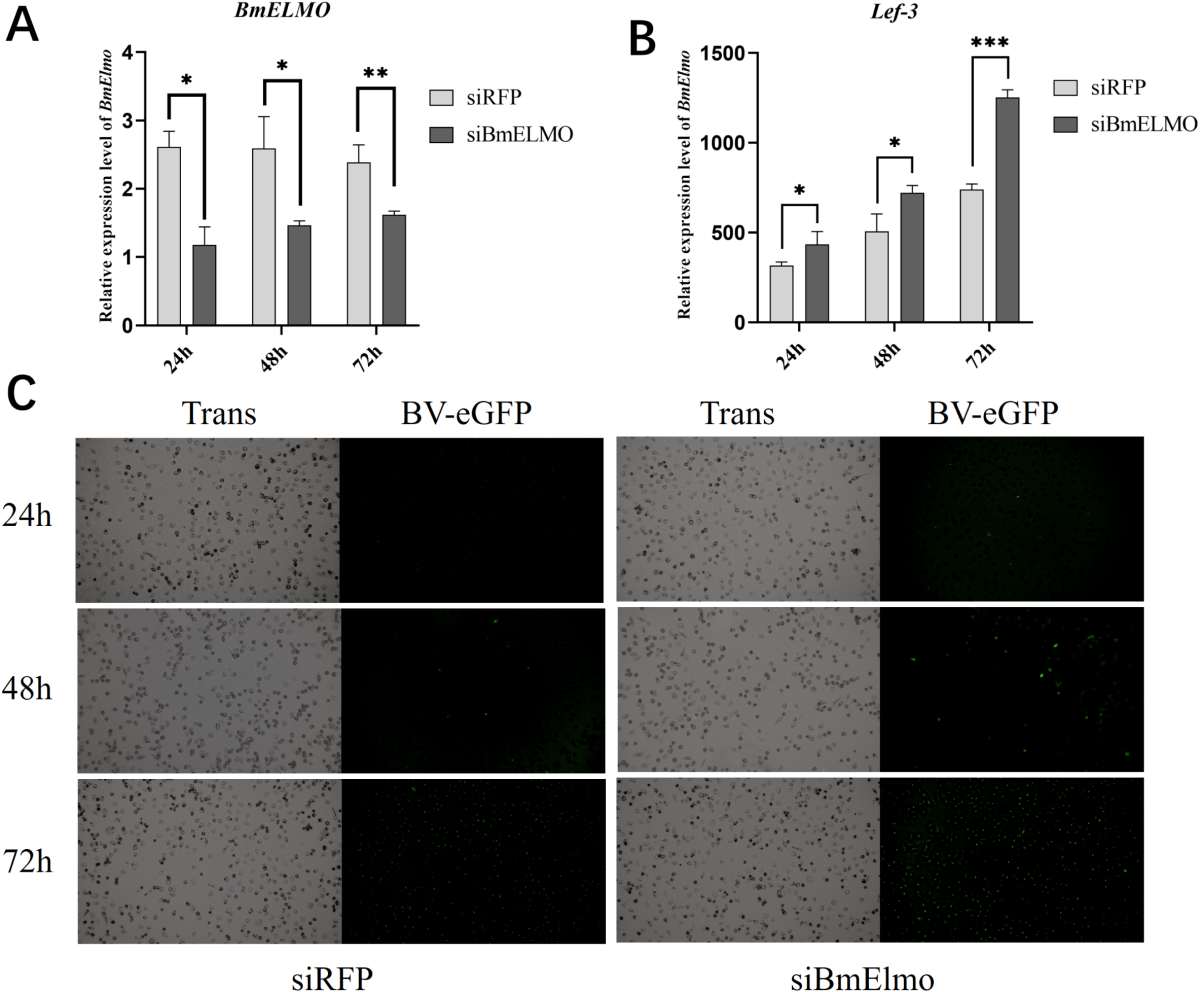
Analysis of AcMNPV proliferation in BmN cells at different times after *BmElmo* knockdown. **(A)** *BmElmo* expression at various times following siRNA transfection. **(B)** Expression of the viral gene *lef3* 24, 48, and 72 h after RNAi. **(C)** eGFP fluorescence signal at 24, 48, and 72 h after RNAi (the scale bar represents 100 μm). *BmGAPDH* was used to normalize the data that were presented as the mean ± standard error of three replicates. Asterisks represent significant differences. *P < 0.05, **P < 0.01, and ***P < 0.001.

The expression level of *BmElmo* has been knocked down in BmN cells by transfecting siRNA which target to its functional domain CED-12 (siBmElmo). Subsequently, these cells were infected with AcMNPV 24 h after transfection. *BmElmo* expression was significantly decreased in the siBmElmo group 24, 48, and 72 h after transfection (**Figure 5A**). AcMNPV infection was observed by fluorescence microscopy at 24, 48, and 72 h post-infection. The virus fluorescence signal rose significantly at 48 and 72 h after infection (**Figure 5C**). To validate the above results, the relative expression of *lef3*, a viral single-strand DNA-binding protein, was detected at 24, 48, and 72 h after AcMNPV infection of BmN cells. *lef3* relative expression was markedly higher in the siBmElmo group compared with the control group (**Figure 5B**).

### 3.5 *BmElmo* overexpression promotes BV-eGFP infection *in vitro*

**Figure 6:**
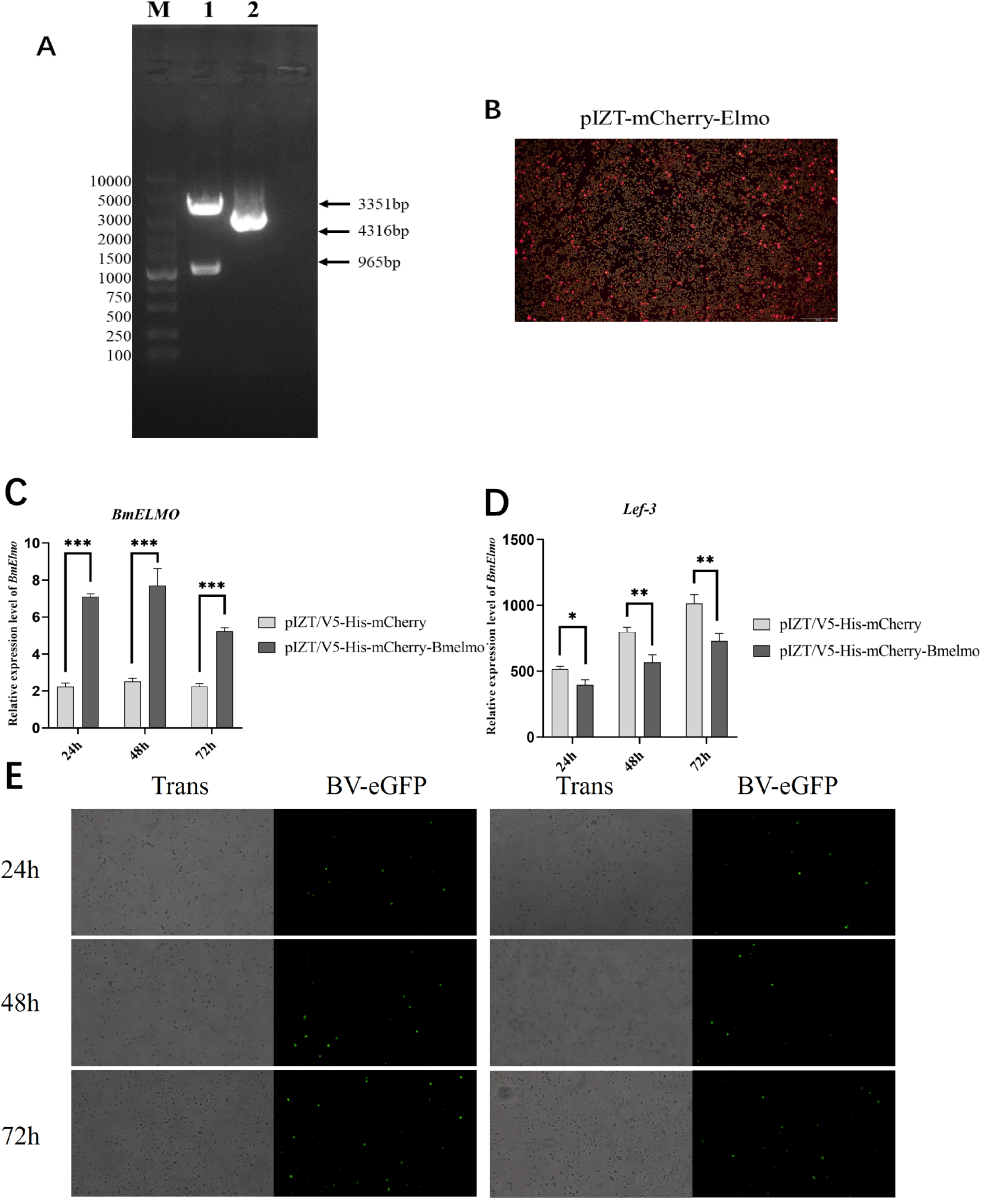
Analysis of AcMNPV proliferation in BmN cells at different times after *BmElmo* overexpression. **(A)** Verification of the pIZT/V5-His-mCherry-BmElmo vector by double restriction enzyme digestion. M, molecular weight of DNA. **(B)** Detection of the pIZT/V5-His-mCherry-BmElmo fluorescence signal in BmN cells (the scale bar represents 100 μm). **(C)** Expression of *BmElmo* 24, 48, and 72 h after *BmElmo* overexpression. **(D)** Expression of the viral gene *lef3* at different times after *BmElmo* overexpression. **(E)** eGFP fluorescence signal 24, 48, and 72 h after *BmElmo* overexpression (the scale bar represents 100 μm). *BmGAPDH* was used to normalize the data that were presented as the mean ± standard error of three replicates. Asterisks represent significant differences. *P < 0.05, **P < 0.01, and ***P < 0.001.

The recombinant plasmid pIZT/V5-His-mCherry-BmElmo was constructed to further investigate *BmElmo*’s role in the AcMNPV response (**Figure 6A**) and transfected BmN cells with it to overexpress *BmElmo* (**Figure 6B**). *BmElmo* expression was detected by RT-qPCR following transfection with the recombinant plasmid. *BmElmo* expression increased significantly at different times after transfection (**Figure 6C**), indicating *BmElmo* was overexpressed successfully in BmN cells. Moreover, the green fluorescence signal of AcMNPV was lower 48 and 72 h after *BmElmo* overexpression compared with the control group (**Figure 6E**). Additionally, *lef3* expression was significantly lower after *BmElmo* expression compared with the control group (**Figure 6D**).

### 3.6 *BmElmo* plays a vital role in regulating the JNK pathway

**Figure 7:**
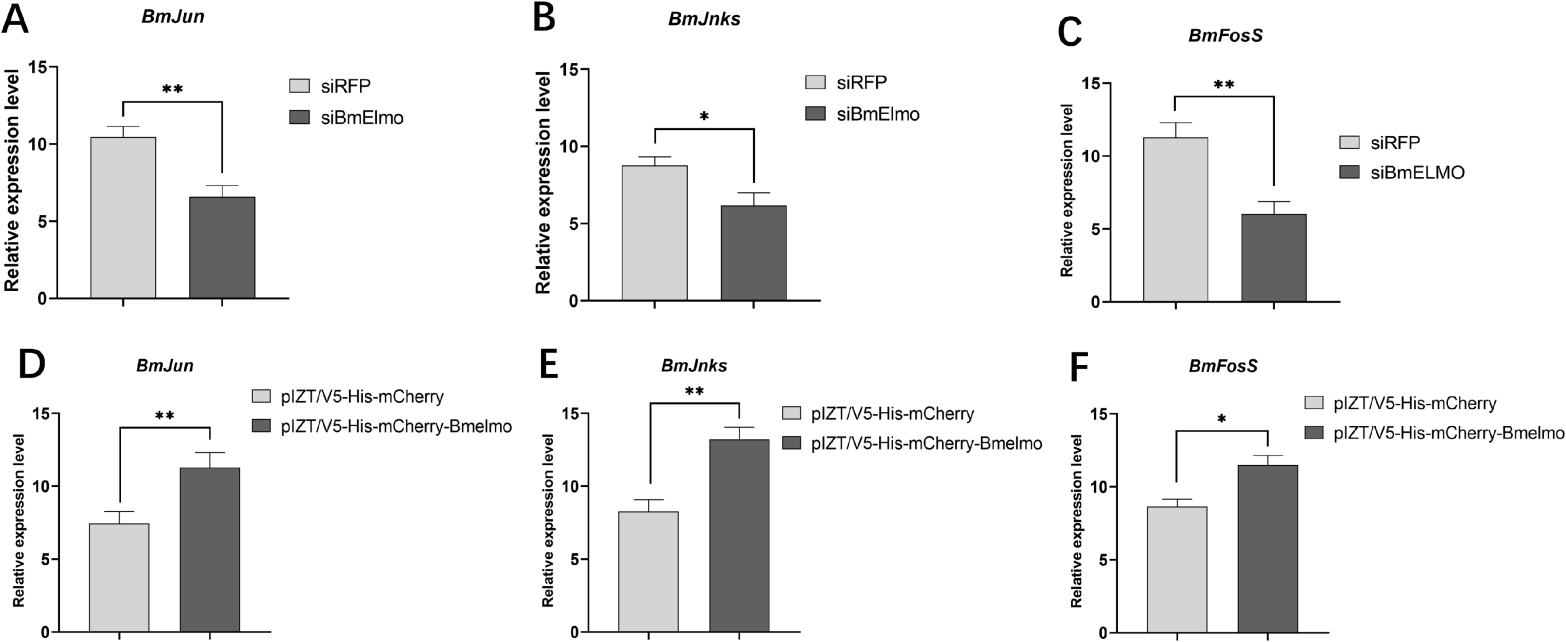
The relative expression of genes related to the JNK pathway in BmN cells 48 h after *BmElmo* knockdown or overexpression based on RT-qPCR. The relative expression levels of **(A)** *BmJun*, **(B)** *BmJnks*, and **(C)** *BmFosS* in BmN cells 48 h after *BmElmo* knockdown. The relative expression levels of **(D)** *BmJun*, **(E)** *BmJnks*, and **(F)** *BmFosS* in BmN cells 48 h after *BmElmo* overexpression. *BmGAPDH* was used to normalize the data that were presented as the mean ± standard error of three replicates. Asterisks represent significant differences. * *P* < 0.05, ** *P* < 0.01.

The pathway by *BmElmo* in BmN cells mediates the response to AcMNPV infection was investigated. Specifically, the expression of genes that encode JNK pathway members and that participate in phagocytosis was examined 48 h after knockdown and overexpression of *BmElmo*. The JNK pathway–related genes BmJun, BmJnks, and BmFosS showed significantly reduced expression after *BmElmo* knockdown (**Figures 7A–C**) and significantly increased expression after *BmElmo* overexpression (**Figures 7D–F**). However, expression of the phagocytosis-related genes BmActinA1 and BmTet did not change significantly after *BmElmo* knockdown or overexpression of (**Supplementary Figure S1**).

## 4 Discussion

AcMNPV can infect more than 30 species of lepidopteran insects (18). Given its wide host range, it is used as a biopesticide in agricultural pest control. However, the resistance of pests to AcMNPV reduces its biocontrol ability. Numerous silkworm species can be infected with AcMNPV through subcutaneous injection. The AcMNPV-sensitive p50 strain and the AcMNPV-resistant C108 strain have been identified by our group (19). After AcMNPV injection, transcriptome analysis revealed that the p50 and C108 strains had significantly different *BmElmo* expression (16, 20). Based on these findings, *BmElmo* seems to have an important role in response to AcMNPV and confirmed in this study.

### 4.1 Sequence characterization and expression profile of B*mElmo* shows its important role in immunity

The Elmo protein sequences from different species was compared and it was found that the *BmElmo* amino acid sequence has been highly conserved, especially its functional domain CED-12 (**Figure 1**). Moreover, CED-12 has been highly phylogenetically conserved among Lepidoptera (**Figure 2**). CED-12 is involved in phagocytosis and apoptosis (21, 22), which provides a valuable clue for detecting the mechanism of *BmElmo* in response to AcMNPV infection. Silkworm larvae at 5th instar is the fastest period of weight gain that body weight increases more than 15-time compared with 1st instar (23). The very high *BmElmo* expression in 1st and 5th instars indicates that it plays a key role in silkworm development (**Figure 3A**). The tissue expression profiles showed that it is highly expressed in ovaries and testis (**Figure 3B**), indicating that it is involved in gonad development, which also corresponds to its high expression during the pupal stage (**Figure 3A**). Of note, the gonads of silkworms are in a primitive stage during the larval period and undergo several maturation steps during the pupal stage. Additionally, *BmElmo* expression was markedly higher compared with the control group in most of the analyzed tissues (**Figure 4**), indicating that *BmElmo* is an important gene in silkworm immunity.

### 4.2 *BmElmo* plays a vital role in resistance to AcMNPV infection *in vitro*

The role of *BmElmo* in the response of BmN cells to AcMNPV infection was further investigated by knocking down and overexpressing this gene. Compared with the control, virus replication increased significantly following *BmElmo* knockdown (**Figure 5**), but decreased significantly after *BmElmo* overexpression (**Figure 6**). These findings demonstrate that *BmElmo* plays a critical inhibitory role in AcMNPV infection of BmN cells.

### 4.3 Immunization of *BmElmo* against AcMNPV may be associated with activation of the JNK pathway

The Elmo protein participates in cellular processes such as cell migration, immunity, and phagocytosis by regulating the downstream RhoA/Rac1 (14), JNK (24) and phagocytosis pathways (6). However, prior to the present study, there had been no reports on which pathways Elmo regulates in the silkworm. In insects, the JNK pathway is closely related to infection of hosts by viruses and other microorganisms (25, 26). In silkworms, reactive oxygen species–mediated JNK phosphorylation is involved in the regulation of *Bombyx mori* nucleopolyhedrovirus (BmNPV) proliferation (27). Phagocytosis is a conserved evolutionary process wherein hemocytes identify, engulf, and ultimately dispose of apoptotic cells and invading microbial pathogens (28, 29). The relative expression of key genes involved in these processes was examined after *BmElmo* knockdown and overexpression. The relative expression of JNK pathway members, *BmJun*, *BmJnks*, and *BmFosS*, changed significantly after *BmElmo* knockdown or overexpression (**Figure 7**). However, the relative expression of the phagocytosis-related genes *BmActinA1* and *BmTet* did not change when *BmElmo* was knocked down or overexpressed (**Supplementary Figure S1**). Hence, the *BmElmo* protein exerts its effects through the JNK pathway, but it is not clear whether the JNK pathway directly affects AcMNPV infection.

Our results indicate that the expression of *BmElmo* can enhance the ability of silkworms to baculoviruses infection, which might be related to the activation of JNK phosphorylation (27, 30), yet this point still needs to be further addressed.

## Data Availability

The original contributions presented in the study are included in the article/**Supplementary Material**. Further inquiries can be directed to the corresponding author.
