# Supplementary figures and images for: BmElmo is a factor for inhibiting Autographa Californica nucleopolyhedrovirus infection in silkworm, Bombyx mori
Source: Front Immunol. 2025 Apr 2;16:1495672. doi: 10.3389/fimmu.2025.1495672 (PMC11999931; doi:10.3389/fimmu.2025.1495672)

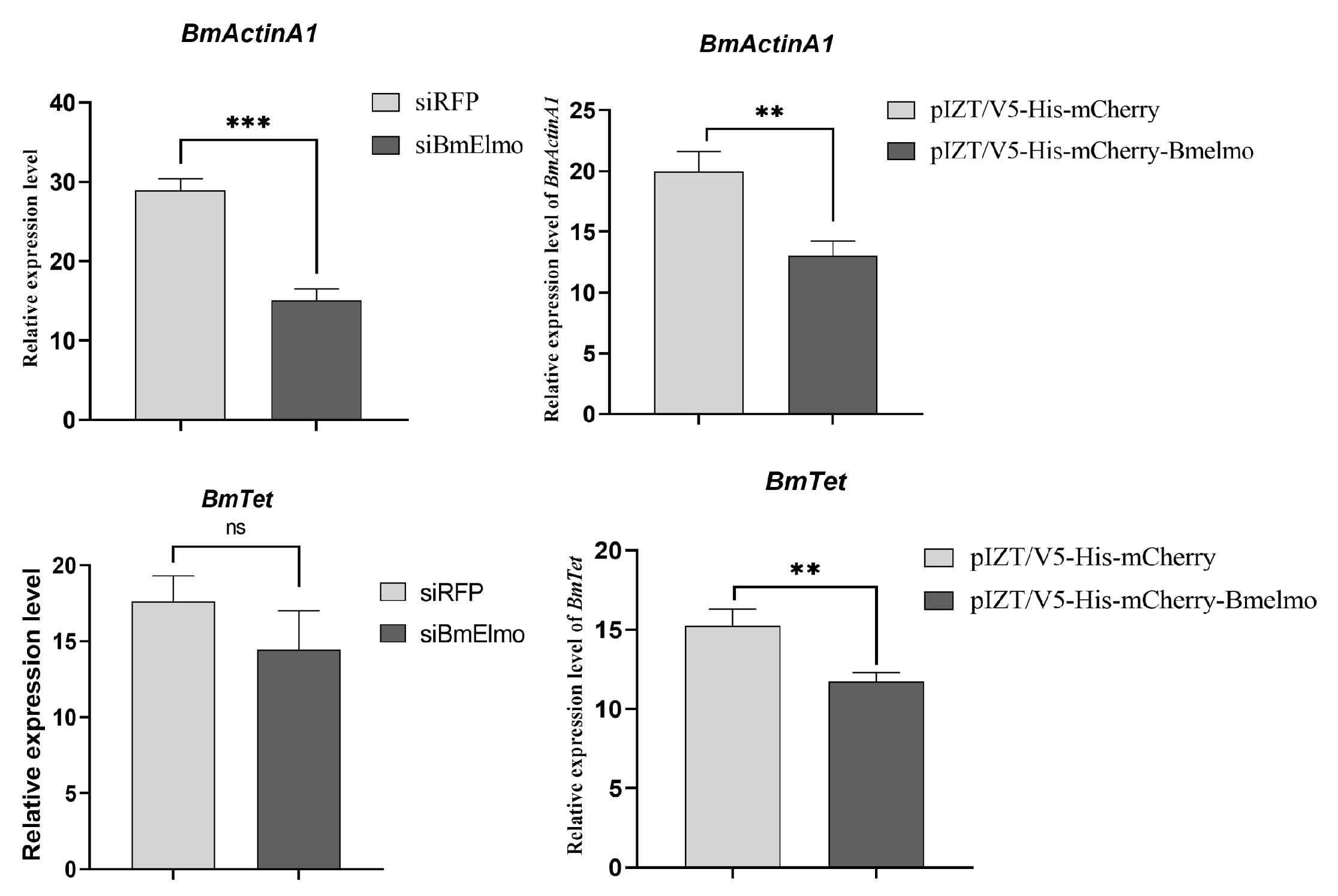

Supplement: Supplementary file 1 [file Image1.tif]
